# Microbiomic and Metabolomic Insights into the Roles of Hydrolysable Versus Condensed Tannins on the Growth Performance, Nutrient Digestion, and Rumen Fermentation in Liaoning Cashmere Goats

**DOI:** 10.3390/microorganisms13040933

**Published:** 2025-04-17

**Authors:** Xin Zhu, Xingtang Dou, Tingting Su, Lisha Ye, Lu Zhang, Haiying Liu, Di Han

**Affiliations:** 1 College of Animal Science and Veterinary Medicine, Shenyang Agricultural University, Shenyang 110866, China; 2 Liaoning Cashmere Goat Breeding Farm Co., Ltd., Liaoyang 111000, China; 3 Liaoning Agricultural Development Service Center, Shenyang 110033, China; 4 State Key Laboratory of Animal Nutrition, College of Animal Science and Technology, China Agricultural University, Beijing 100193, China

**Keywords:** goat, tannin, growth performance, rumen fermentation, microbiota

## Abstract

This study was conducted to compare the effects of hydrolysable versus condensed tannins on growth performance, nutrient digestion, and rumen fermentation in goats. A total of 27 Liaoning cashmere goats with similar initial body weights were randomly distributed into three groups: a basal diet, a basal diet +0.5% tannic acid (hydrolysable tannin, HT), or a basal diet +0.5% quebracho tannin (condensed tannin, CT), respectively. Compared to the control group, HT showed a decreased (*p* < 0.05) feed intake, while CT showed an increased (*p* < 0.05) feed intake and body weight gain. Digestibility of dry matter, crude protein, neutral detergent fiber, and acid detergent fiber did not differ (*p* > 0.05) among groups. The HT group showed lower pH and acetic acid concentration in the rumen (*p* < 0.05), while the CT group showed a decrease (*p* < 0.05) in the abundances of *Verrucomicrobia* and *Methanobrevibacter*. Supplementation of CT decreased (*p* < 0.05) the levels of dihydrouracil, xanthosine, alpha-D-mannose, and L-tryptophan, while HT increased (*p* < 0.05) these metabolites. In conclusion, this study suggested that responses of ruminal microbiota and metabolite profiles to the type of tannins varied, and CT displayed a more positive effect on the growth performance of goats than HT when supplemented at the same level in the diets.

## 1. Introduction

Since the 1940s, antibiotics have been widely applied in livestock and poultry because of their effectiveness in treating disease, protecting health, and promoting growth [1]. The increasing demand for animal protein foods prompts the overuse and/or abuse of antibiotics in the feeding process, consequently leading to antibiotic residues and resistance, and causing enormous threats to human health and environmental protection [2]. The use of antibiotics has gradually been banned worldwide in recent years. Global efforts to phase out the routine use of antibiotics and to develop and utilize new sustainable, residue-free alternatives to antibiotics are more pressing than ever before [3,4].

Plant extracts, including polyphenols, essential oils, alkaloids, flavonoids, and polysaccharides, are natural bioactive compounds that possess sustainable, safe, and efficient characteristics and have been widely used in animal production [5]. Tannins, belonging to polyphenols, are widely distributed in plant tissues and are considered to be an antinutritional factor because they form complexes with proteins, polysaccharides, digestive enzymes, and metal ions, which hampers the digestion and absorption of nutrients by animals and is even considered to be toxic [6]. Many recent studies have shown that supplementation with tannins at moderate concentrations in the diet could improve anti-inflammatory, antioxidant, and antibacterial properties, while not affecting or enhancing the growth performance in pigs and broilers [7,8,9,10].

Hydrolysable tannin (HT) and condensed tannin (CT) are the two main groups of tannins in plants. HT has a polyol core (commonly D-glucose) esterified with phenolic acids (mainly gallic or hexahydroxy diphenic acid) with molecular weights from 500 to 3000 Da and is susceptible to hydrolysis by acids, bases, or esterases, which can be easily degraded and absorbed in the digestive tract and even cause potential toxic effects in herbivores [11]. CT is composed of flavan-3-ol units, including catechin, epicatechin, gallocatechin, and epigallocatechin, and forms complex oligomeric or polymeric flavonoids with higher molecular weights ranging from 1000 to 20,000 Da, and can only be depolymerized by strong oxidative and acidic hydrolysis [12]. For instance, tannins extracted from chestnut wood (*Castanea sativa* Miller) and quebracho (*Schinopsislorentzii*) are examples of HT and CT, respectively [13]. Tannins, especially CT, are widely distributed in nutritionally important forages, trees, shrubs, and legumes, which are commonly consumed by ruminants [11]. Ruminants have developed their own characteristic rumen, which is the biggest internal organ and the main site where feed is digested and fermented by its symbiotic microbes, producing fermentation end products such as energy-accessible volatile fatty acids (VFAs) to support growth and development [14,15]. Feeding ruminants low or moderate levels of tannin (<5%) has been demonstrated to modulate rumen fermentation, reduce energy loss, and maintain health while not producing negative effects on the growth performance and nutrient digestibility [16]. However, it has been suggested that excessive addition of tannin (≥5%) in the diet could hamper the growth and decrease feed intake, thus leading to inferior production performance in ruminants [17]. In addition, the effect of tannins on ruminants is dependent on the type of tannins consumed. However, to date, there is no clear understanding of the mechanism of tannins on the growth and rumen fermentation in ruminants. In this study, we used the microbiome and metabolome technologies to investigate the effects of different types of tannins (hydrolysable vs. condensed) on growth and rumen fermentation and elucidate the mechanism in goats.

## 2. Materials and Methods

All animal experimental procedures were reviewed and approved by the Animal Care and Use Committee of Shenyang Agricultural University (approval number 202203090).

### 2.1. Animals, Experimental Design, and Management

A total of 27 one-year-old healthy Liaoning cashmere female goats with body weights (BW) of 32.59 ± 2.15 kg were selected and randomly assigned to 3 dietary groups. Each group had 9 replicates, and each replicate (goat) was reared in a metabolism cage (1.2 m length × 0.6 m width × 1.6 m height). The goats were fed a basal diet (control), a basal diet +0.5% tannic acid (HT), and a basal diet +0.5% quebracho tannin (CT), respectively. After a two-week adaptation period, the goats were given their individual diets for 6 weeks. The basal diet was formulated to meet the nutrient requirements of goats in the growing stage according to the China standard of nutrient requirements of cashmere goats (NT/T 4048–2021) [18], as shown in Table 1. The diets were fed in the morning (0800) and afternoon (1700), and diets and clean water were given ad libitum. All goats were dewormed before the experiment and reared according to the routine management protocols of Liaoning cashmere goats.

### 2.2. Intake and Performance

During the feeding period, the feed intake (FI) and the feed orts of the goats in each cage were recorded daily, while the individual body weight was weighed weekly. The ratio of feed to gain (F/G) was calculated as average daily feed intake (ADFI) divided by average daily gain (ADG).

### 2.3. Nutrient Digestion

From days 38 to 42 of the feeding period, a total collection of feces was performed daily. The daily feces output was weighed, and 10% of the total output from each replicate was pooled and then stored at −20 °C for chemical analysis.

### 2.4. Rumen Fluid Collection

On the last day of the experiment, the rumen fluid was collected using an oral-stomach probe that contained a metal head acting as a sieve and a manual pump with a glass container. Approximately 100 mL of rumen fluid was collected and filtered with 4 layers of sterile gauze. The pH value was measured immediately, after which the filtered rumen fluid was divided into 10 mL sterile tubes and then stored at −80 °C for further analysis.

### 2.5. Laboratory Analysis

Feed and feces samples were analyzed for dry matter (DM, Method 934.01), crude protein (CP, Method 976.05), ether extract (EE, Method 920.39), ash (Method 942.05), crude fiber (CF, Method 978.10), neutral detergent fiber (NDF, Method 2002.04), and acid detergent fiber (ADF, Method 973.18) according to the procedures of AOAC International [19].

For the determination of NH_3_-N and volatile fatty acid (VFA) concentrations, 10 mL of collected rumen fluid was centrifuged at 4000× *g* for 10 min. A 2 mL of supernatant was collected and mixed with 8 mL of 0.2 mol/L hydrochloric acid for the colorimetric analyses according to the method described before [20] to determine NH_3_-N concentration. Another 1 mL of supernatant was collected and transferred into a 1.5 mL centrifuge tube. A 0.2 mL 1% crotonic acid was added to the tube, and the mixture was vortexed and then ice-bathed for 30 min. After centrifuging at 12,000× *g* for 10 min, the supernatant was collected and filtered through a 0.22 µm filter. The VFA concentrations in the filtrate were determined by gas chromatography (7890B, Agilent Technologies, Santa Clara, CA, USA) with a flame ionization detector and a fused silica column (FFAP 30 m × 0.25 mm × 0.25 µm, DiKMA Technology Co., Ltd., Beijing, China). The injector and detector temperatures were set at 220 °C and 250 °C, respectively. The column temperature increased from 80 °C to 150 °C at 10 °C/min, and further increased from 150 °C to 180 °C at 15 °C/min, which was held for 1 min. The injection volume of the sample was 1.0 µL.

### 2.6. Microbial DNA Extraction and 16S rRNA Gene Sequencing

A 10 mL sample of filtered rumen fluid was centrifuged at 10,000× *g* for 10 min at 4 °C, and 200 mg of the pellet was obtained for DNA extraction. The total DNA was extracted through the CTAB-based buffer plus bead beating, as described previously [21]. The quality of the extracted DNA was examined using 1% agarose gel electrophoresis, and the yield of the extracted DNA was quantified using a Qubit 3.0 fluorometer (Invitrogen, Carlsbad, CA, USA). The extracted DNA was then normalized to 20 ng/µL with double-distilled water as a template. The V3-V4 regions of 16S rRNA genes were amplified using the forward primer 338F (5’-ACTCCTACGGGAGGCAGCA-3’) and the reverse primer 806R (5’-GGACTACHVGGGTWTCTAAT-3’). The PCR amplified system (25 µL) was composed of 5 µL of 5× reaction buffer, 5 µL of 5× GC buffer, 2 µL of 2.5 mM dNTP, 1 µL of forward primer, 1 µL of reverse primer, 2 µL of DNA template, 8.75 µL of double-distilled water and 0.25 µL of high-fidelity DNA polymerase by following the manufacturer’s instructions (M0491L, NEB Inc., Ipswich, MA, USA). The PCR amplification procedure was performed as follows: 98 °C initial denaturation for 2 min, followed by 30 cycles of 98 °C denaturation for 15 s, 55 °C annealing for 30 s, and 72 °C extension for 30 s, and a final cycle of 72 °C extension for 5 min. The PCR amplification products were purified using 2% agarose gel electrophoresis, and the concentrations of amplicons were quantified using a Qubit 3.0 fluorometer (Invitrogen, Carlsbad, CA, USA). The amplicons were pooled in equimolar concentrations to give a final concentration of 20 pM for library construction. The library was then sequenced on an Illumina MiSeq platform (Personalbio Co., Ltd., Shanghai, China).

After sequencing, the adapter sequences from high-throughput raw reads were removed using the cutadapt plugin (v2.3) of QIIME 2 (v2019.4). Sequences were then quality-filtered by denoising, merging, and chimera removal using the DADA2 method [22]. The qualified sequences were re-clustered into operational taxonomic units (OTUs) at a threshold level of 97% sequence identity following the procedure of Vsearch (v2.13.4). The taxonomic classification was completed using the classify-sklearn algorithm [23] and the Greengenes2 database (http://ftp.microbio.me/greengenes_release/, accessed on 14 April 2025). The alpha and beta diversity metrics were estimated using the diversity plugin of QIIME2 (v2019.4).

### 2.7. Rumen Metabolites Extraction and Quantification

Rumen fluid, 0.5 mL, was mixed with 0.5 mL of 80% methanol solution and vortexed for 1 min. The mixture was centrifuged at 12,000× *g* at 4 °C for 10 min, and the supernatant was collected, concentrated, freeze-dried, and then re-dissolved and mixed with 150 µL of 2-chlorine-L-phenylalanine solution (4 mg/kg in 80% methanol). The supernatant was filtered through a 0.22 µm filter membrane, and the filtered fluid was collected for liquid chromatography–mass spectrum (LC-MS) analysis.

A high-performance LC system (Vanquish, Thermo Fisher Scientific, Waltham, MA, USA) equipped with an ACQUITY UPLC HSS T3 column (2.1 × 150 mm, 1.8 µm, Waters, Milford, CT, USA) was used for chromatographic separation. The mobile phase was acetonitrile, and the flow rate was 0.25 mL/min. The column temperature was set at 40 °C, and the injection volume was 2 µL. For detecting metabolites, a Thermo Q Exactive Focus MS system (Thermo Fisher Scientific, Waltham, MA, USA) equipped with an electrospray ionization (ESI) source was used. The method of XCMS (various forms (X) of chromatography mass spectrometry) was adopted for automatic processing of data for feature detection and calculation of chromatographic peak areas [24]. Metabolite identification was performed using the HMDB 5.0 (https://hmdb.ca/, accessed on 14 April 2025) [25], MassBank (https://massbank.jp/, accessed on 14 April 2025) [26], and LipidMaps (https://www.lipidmaps.org/, accessed on 14 April 2025) [27] databases. Principal components analysis (PCA) and orthogonal partial least squares discriminant analysis (OPLS-DA) were used to visualize a classification model and provide quantitative information for discriminating metabolites using the R package (v3.0.2). Metabolites with both *p* < 0.05 and variable importance in projection (VIP) scores higher than 1.0 were considered statistically significant. The functional pathway enrichment and topology analysis of significantly varied metabolites was conducted using a MetaboAnalyst 6.0 platform (https://www.metaboanalyst.ca/, accessed on 14 April 2025) [28] and visualized by the Mapper plugin of the Kyoto Encyclopedia of Genes and Genomes (KEGG, http://www.kegg.jp/, accessed on 14 April 2025).

### 2.8. Statistical Analyses

In this present study, each goat was used as an experimental unit, and the SPSS 22.0 software (SPSS Inc., Chicago, IL, USA) was used to analyze the data. The following model was used:

Y_ij_ = µ + X_i_ + ε_ij_,

where Y_ij_ is the dependent variable, µ is the overall mean, X_i_ is the fixed effect of treatment (i = 0 and 0.5% tannin), and ε_ij_ is the random residual error. If the variables showed a normal distribution, one-way analysis of variance (ANOVA) was used to analyze the data, and a Duncan’s multiple range test was performed to adjust for multiple comparisons. The variables that showed a non-normal distribution were analyzed using a non-parametric factorial Kruskal–Wallis sum–rank test. Statistical significance was declared at *p* < 0.05.

## 3. Results

### 3.1. Growth Performance

As shown in Table 2, dietary supplementation with tannins affected BW gain and DM intake (*p* < 0.05), and compared to the control and HT group, the CT group showed the highest BW gain and DM intake (*p* < 0.05). There were no significant differences in initial BW, final BW, and F/G among groups (*p* > 0.05).

### 3.2. Nutrient Digestibility

Results from Table 3 showed that there were no significant differences in the digestibilities of DM, CP, NDF, and ADF among groups (*p* > 0.05).

### 3.3. Rumen Fermentation

Dietary supplementation with tannins affected the pH and NH_3_-N concentration of the rumen (*p* < 0.05) (Table 4). Compared to the control and CT groups, the HT group showed a lower pH (*p* < 0.05). Although there were no significant differences in the NH_3_-N (*p* = 0.05) and total VFA concentrations among groups (*p* > 0.05), the proportions of acetic acid and valeric acid were affected by the dietary treatments (*p* < 0.05). The proportion of acetic acid in the HT group was lower than that in the control and CT groups (*p* < 0.05), while the proportion of valeric acid in the CT group was higher than that in the control and HT groups (*p* < 0.05). There were no significant differences in the proportion of propionic acid, butyric acid, isobutyric acid, isovaleric acid, and acetic/propionic ratio among groups (*p* > 0.05).

### 3.4. Microbiota Composition

As shown in Table 5, indices of Shannon and Simpson were not affected by supplementation with tannins in the diet (*p* > 0.05). However, there was an increasing trend in the Chao1 index in the tannin supplementation treatments (*p* < 0.10), indicating increased microbial richness of the rumen. The principal coordinates analysis (PCoA) with Bray–Curtis distance results showed that the HT or CT groups separated from the control group (Supplementary Figures S1 and S2), indicating that the tannin supplementation changed the microbiota composition of the rumen. At the phylum level, a total of 28 phyla were identified, but only 3 phyla (Bacteroidetes, Firmicutes, and Verrucomicrobia) had a relative abundance of >1% in all groups (Table 6). Compared to the control group, the relative abundance of Bacteroidetes in the HT and CT groups was higher (*p* < 0.05), while that of Verrucomicrobia in the CT group was lower than in the control and HT groups (*p* < 0.05). At the genus level, a total of 365 genera were identified in all groups, and the top 15 genera with relative abundance of >0.1% are presented in Table 7. Among these genera, *Prevotella*, *Rikenellaceae RC9*, *Bacteroidales F082*, *Ruminococcaceae* UCG, *Kiritimatiellae WCHB1-41*, and *Christensenellaceae* R7 were dominant in the rumen, with the relative abundance of >1%. The relative abundance of *Bacteroidales* RF16 in the HT group was higher than that in the control and CT groups (*p* < 0.05), while the relative abundance of *Bacteroidales* BS11 was higher in the CT group than that in the control and HT groups (*p* < 0.05). In addition, supplementation with HT increased the relative abundance of *Prevotella* and *Erysipelotrichaceae* UCG and decreased that of *Rikenellaceae* RC9, *Ruminococcaceae* UCG, and *Halomonas* in a trend (*p* < 0.10), while supplementation with CT decreased the relative abundance of *Kiritimatiellae WCHB1-41* in a trend (*p* < 0.10). The linear discriminant analysis (LDA) effect size (LEfSe) analysis indicated that *Halomonas* and *Streptococcus* were dominant in the control group, while supplementation with HT increased the relative abundance of *Prevotella* and *Bacteroidales* RF16, and CT increased the relative abundance of *Bacteroidales* BS11 (Figure S3). The methanogen variation profiles of the rumen were also analyzed, as shown in Table 8. Compared to the control and HT groups, the CT group had a lower relative abundance of *Methanobrevibacter* and a higher relative abundance of *Candidatus Methanoplasma* (*p* < 0.05).

### 3.5. Metabolite Profiles

**Figure 1:**
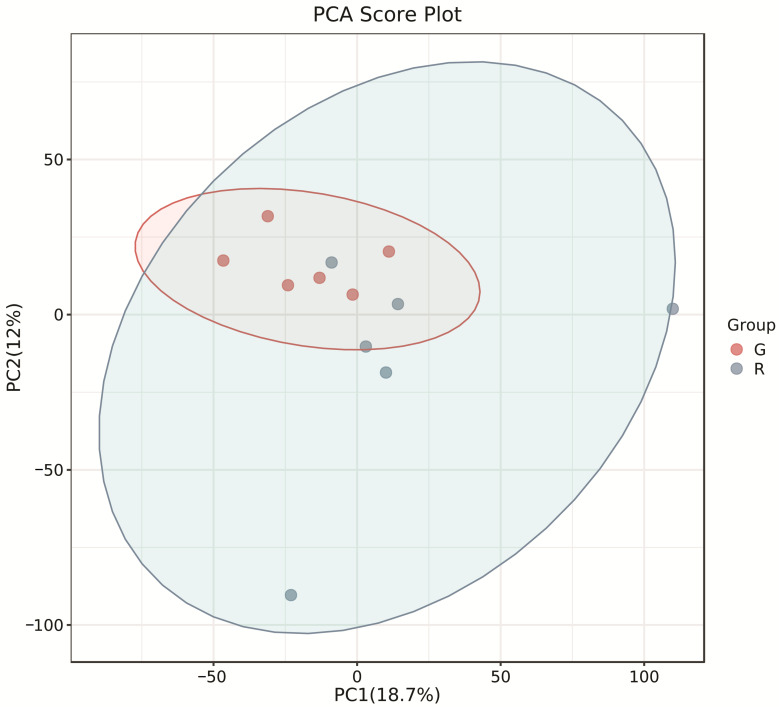
Principal component analysis (PCA) of rumen metabolites from the control and HT groups in Liaoning cashmere goats. G, control group; R, HT group.

**Figure 2:**
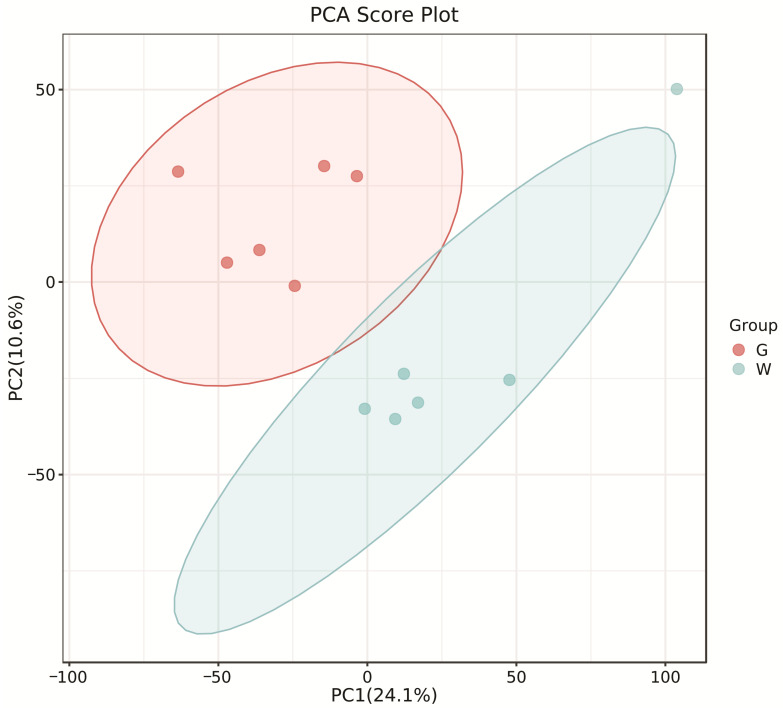
Principal component analysis (PCA) of rumen metabolites from the control and CT groups in Liaoning cashmere goats. G, control group; W, CT group.

**Figure 3:**
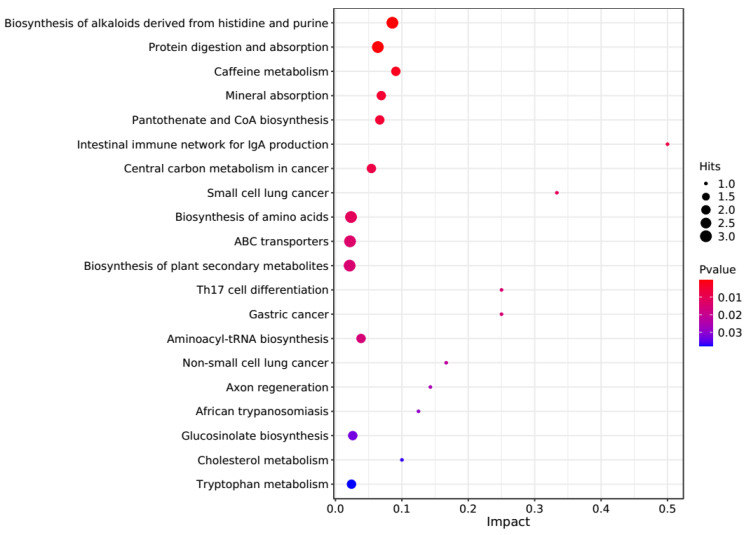
Kyoto Encyclopedia of Genes and Genomes (KEGG) pathway enrichment analysis of differentially altered metabolites in the rumen between the control and HT groups. Larger bubbles indicate more significant altered metabolites in the related pathway, whereas redder bubbles indicate a greater contribution of detected altered metabolites to the related pathway.

**Figure 4:**
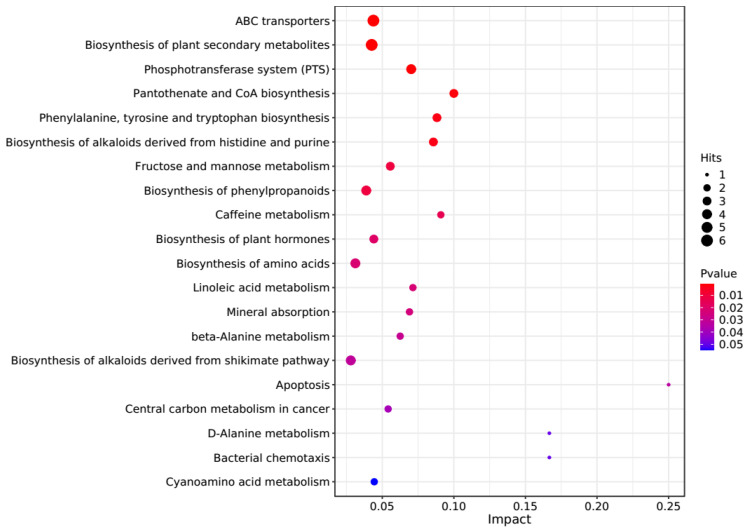
Kyoto Encyclopedia of Genes and Genomes (KEGG) pathway enrichment analysis of differentially altered metabolites in the rumen between the control and CT groups. Larger bubbles indicate more significant altered metabolites in the related pathway, whereas redder bubbles indicate a greater contribution of detected altered metabolites to the related pathway.

The PCA score plots showed no clear distinction between the control and HT groups (Figure 1), but presented the most dramatic change in the metabolite profile between the control and CT groups (Figure 2). A total of 1865 metabolites were detected in all groups. There were 23 differentially expressed metabolites between the control and HT groups, among which 11 metabolites were upregulated and 12 were downregulated, and 52 differentially expressed metabolites between the control and CT groups, among which 30 metabolites were upregulated and 22 were downregulated (Table S1). Significantly different metabolic pathways were observed in this study. According to a KEGG analysis, the main pathways that the altered metabolites were associated with biosynthesis of alkaloids derived from histidine and purine, protein digestion and absorption, biosynthesis of amino acids, ABC transporters, and biosynthesis of plant secondary metabolites in the HT group, as shown in Figure 3, while those in the CT group were ABC transporters, biosynthesis of plant secondary metabolites, phosphotransferase system, biosynthesis of phenylpropanoids, and biosynthesis of amino acids, as shown in Figure 4.

### 3.6. Correlations Between the Microbiome and Metabolome

**Figure 5:**
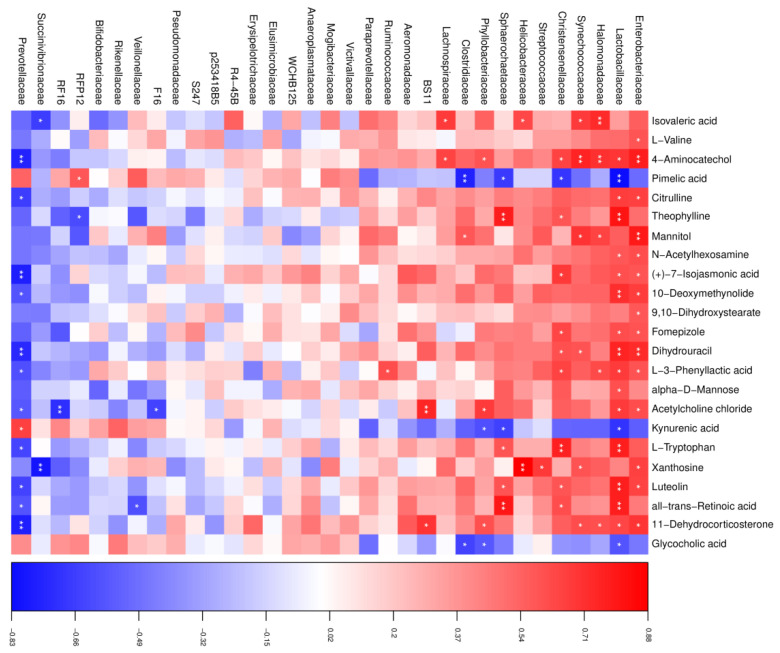
Correlation analysis between the altered rumen microbial and metabolites in the HT group. Red indicates a positive correlation, and blue indicates a negative correlation. * represents *p* < 0.05 and ** represents *p* < 0.01.

To reveal functional relationships between the rumen microbiota and metabolites, Pearson’s correlation analysis was performed. As shown in Figure 5, in the HT group, a total of 96 significant correlations were recognized, among which 69 correlations were positive, and 27 were negative. The abundance of *Enterobacteriaceae*, *Lactobacillaceae*, and *Prevotellaceae* was most correlated with the changed metabolites, in which both *Enterobacteriaceae* and *Lactobacillaceae* were positive and *Prevotellaceae* was negative, except for pimelic acid, kynurenic acid, and glycocholic acid, which showed opposite correlations. In addition, dihydrouracil had a positive correlation with *Enterobacteriaceae* and *Lactobacillaceae* and a negative correlation with *Prevotellaceae*. L-tryptophan was found to be positively correlated with *Lactobacillaceae*. We also found that acetylcholine chloride was positively correlated with *Bacteroidales* BS11 and negatively correlated with *Bacteroidales* RF16.

**Figure 6:**
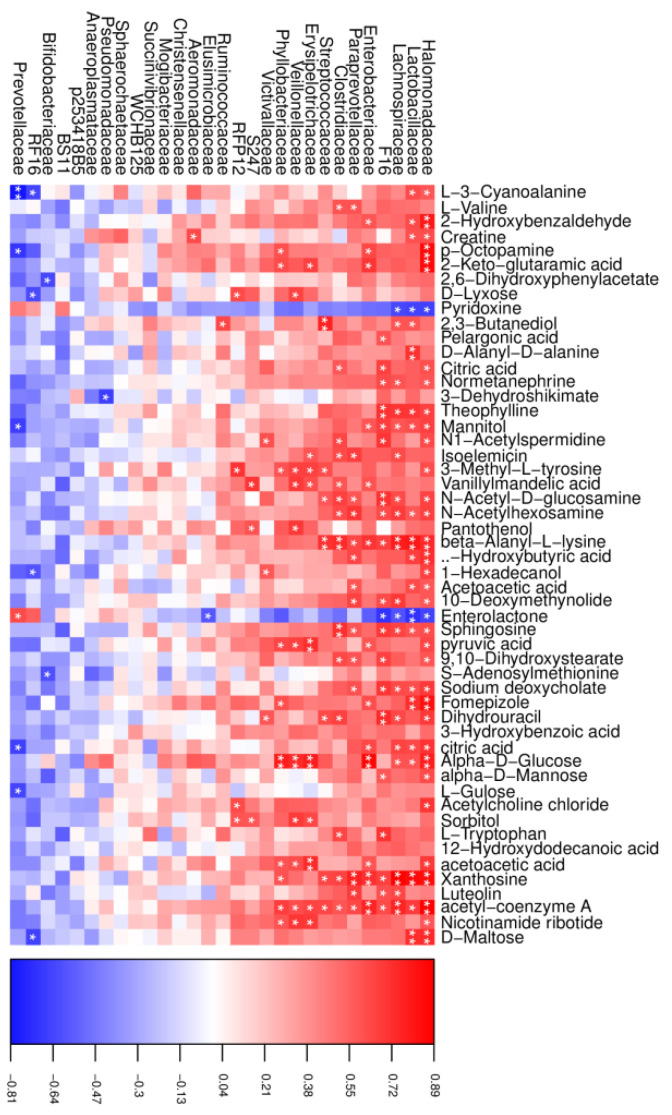
Correlation analysis between the altered rumen microbial and metabolites in the CT group. Red indicates a positive correlation, and blue indicates a negative correlation. * represents *p* < 0.05 and ** represents *p* < 0.01.

In the CT group, a total of 195 significant correlations were recognized, among which 175 correlations were positive, and 20 were negative (Figure 6). *Halomonadaceae*, *Lactobacillaceae*, and *Prevotellaceae* were the most correlated with the changed metabolites, in which both *Halomonadaceae* and *Lactobacillaceae* were positive and *Prevotellaceae* was negative, except for pyridoxine and enterolactone, which showed opposite correlations. In addition, we also found that xanthosine was positively correlated with the abundance of *Halomonadaceae*, *Lactobacillaceae*, *Lachnospiraceae*, and *Enterobacteriaceae*.

## 4. Discussion

In ruminants, feed intake, nutrient digestion, and rumen fermentation are closely related to the growth performance. Tannin has a bitter taste, and inclusion in the diet could decrease feed intake, but this is not always the case. The effect of tannin on feed intake and weight gain is concentration-dependent, and a low or moderate amount of tannin containing less than 50 g/kg DM in the diet has little or no effect on the feed intake and weight gain of the ruminants [29,30]. In this study, the level of tannin added in the diet was 0.5%, which suggests no negative effect on growth performance. Interestingly, dietary supplementation with CT, compared to HT, increased dry matter intake and weight gain in this study. The mechanism of these different types of tannins on goats may be related to rumen fermentation, and this is discussed below.

Numerous previous studies have indicated varied affinities of tannins for dietary dry matter, fiber, and protein. Generally, tannin modifies nutrient digestibility by affecting the rumen fermentation pattern, as tannin could influence rumen microbiota and the accessibility of rumen microbiota to nutrients, such as protein. However, the negative effect of tannin on nutrient digestibility was concentration-dependent. Bhatt et al. [31] investigated combinations with different HT and CT levels in the diet of sheep and reported that there were no significant differences in the DM, CP, NDF, and ADF digestibilities between the low and medium tannin content diets, similar to our results.

Previous studies have shown that a lower addition level (less than 5%) of tannin in the diet had little or no effect on rumen fermentation parameters [31,32]. Similarly, our study also demonstrated that dietary supplementation with 0.5% CT had no effect on the rumen pH, NH_3_-N, and total VFAs concentrations. Interestingly, in this study, we found that adding 0.5% HT (tannic acid) into the diet decreased the pH of the rumen. An explanation may be that tannic acid is a weak acid with carboxy groups (-COOH), which naturally lowers the pH.

Rumen microbiome plays an important role in the fermentation of fibrous and non-fibrous feedstuffs to yield products such as VFAs, and it is mainly composed of bacteria (>1000 cells/g rumen content) and includes over 200 species [33]. In this study, we found that dietary supplementation with both HT and CT had the ability to increase microbiota richness, indicative of stimulating the growth of bacteria. Bacteroidetes and Firmicutes are the main phyla that are predominant in the rumen bacterial community, and both are main fiber degraders and VFA producers [34]. Similarly, the relative abundance of Bacteroidetes and Firmicutes was over 85% in the rumen, and the abundance of Bacteroidetes was increased in the tannin groups, indicating that dietary supplementation with tannin did not affect the growth of cellulolytic bacteria. Interestingly, we found that supplementation with CT, not HT, decreased the abundance of Verrucomicrobia in the rumen. Verrucomicrobia were reported to produce H_2_ in the rumen and were prevalent in high-methane-yielding animals. This suggests that dietary supplementation with CT may decrease the methane production in the rumen. At the genus level, HT increased the abundance of *Bacteroidales* RF16, and CT increased that of *Bacteroidales* BS11 in this study, indicating that HT and CT have varied abilities to modulate the specific bacterial growth in the rumen. Bacteroidales, belonging to the phylum of Bacteroidetes, are important complex carbohydrate degraders in the host gut. Fonseca et al. [35] and Rabee et al. [36] demonstrated that dietary tannin supplementation significantly enhanced the abundance of Bacteroidetes in the rumen. This selective bacterial stimulation may be attributed to the inhibitory effects of tannins on proteolytic activity in competing bacterial populations. Specifically, tannins penetrate microbial cell walls, interact with structural components, and bind to surface polymers, thereby suppressing rival bacterial growth while promoting the proliferation of specific bacterial taxa [37,38]. The increased prevalence of these fibrolytic bacteria likely enhances ruminal fiber fermentation efficiency, leading to improved nutrient digestibility and subsequent growth performance in ruminants.

It was reported that tannin can be used to mitigate methane (CH_4_) emissions by ruminants, and this mitigating effect depended mainly on the dose and type of tannin added in the diet [31,39]. When the dose was below 4% DM, tannin can produce beneficial effects by improving rumen fermentation and reducing CH_4_ emissions in ruminants [16]. The mechanism of CH_4_ mitigation through the dietary inclusion of tannin was reported to either indirectly reduce rumen fiber fermentation, thus decreasing H_2_ and acetate production, or directly inhibit the growth of methanogens [40]. In this study, we found that dietary supplementation with CT, not HT, decreased the abundance of *Methanobrevibacter* in the rumen, indicating the potential to reduce CH_4_ emissions. This difference in lowering methanogen abundance between CT and HT may result from the binding capacity of the phenolic hydroxyl groups of tannin to macromolecules (proteins, structural carbohydrates, and starch) in the rumen. These complexes render the macromolecules unavailable to rumen microbes, thus reducing microbial growth and decreasing CH_4_ production. In addition, this binding capacity was demonstrated to be positively associated with the molecular weight of tannin, being more effective with higher molecular weights [41].

It has been observed that tannin supplementation in the diet could affect the metabolic profiles in the rumen. This study showed that the total number of upregulated and downregulated metabolites in the CT group was higher than those in the HT group, indicating that CT had a greater ability to alter the metabolite profiles in the rumen of goats. From an overview of metabolite profiles, we found that there was a different regulation mechanism between HT and CT. In this study, dietary supplementation with HT increased the levels of dihydrouracil, xanthosine, alpha-D-mannose, and L-tryptophan in the rumen of goats, while CT decreased these metabolites, indicative of differential regulatory pathways in the nucleotide, carbohydrate, and amino acid metabolisms. Dihydrouracil is an intermediate in the pyrimidine nucleotide metabolism, which can be oxidized into uracil. Some bacteria (such as *Clostridium*) can utilize it to produce β-alanine, carbon dioxide, and ammonia [42]. Xanthosine, a purine nucleoside, is an intermediate of the catabolic pathway of guanosine monophosphate [43]. These nucleosides are key components of the signal transduction pathways involved in the regulation of homeostasis, cell cycle, and secondary metabolite biosynthesis. In ruminants, these metabolites are mainly produced during microbial protein synthesis, and the reduced levels of dihydrouracil and xanthosine in the CT group may account for the decreased microbial activity. D-mannose, an epimer of glucose, is a major component of hemicellulose polysaccharide mannans and plays a crucial role in protein glycosylation [44]. In the rumen, bacteria are the most active complex polysaccharide degraders to produce soluble monomers, and the substrate accessibility by rumen microbiota is fundamental to fiber degradation [15]. In this study, a decreased level of D-mannose in the rumen of CT-fed goats suggested that CT may have a higher ability to impede the binding of microbes to feed particles than HT, thus resulting in decreased hemicellulose degradation. Tryptophan is an essential amino acid that can be used for protein biosynthesis or other key metabolic components, such as serotonin, niacin, and melatonin [45]. In ruminants, tryptophan may be easily degraded in the rumen by microorganisms before it passes to the small intestine for absorption [46]. The end products from ruminal degradation of tryptophan are indole and skatole, which are well-known foul-smelling fecal odorants in livestock and poultry feces [47]. In this study, we found that goats fed with a CT diet had a lower level of L-tryptophan in the rumen than those fed with an HT diet. An explanation for this result may be that dietary supplementation with CT stimulated the tryptophan-metabolizing bacteria growth, such as Bacteroidetes, to degrade tryptophan into indole and its derivatives. Bacteroidetes were increased in the CT group and were previously demonstrated to metabolize tryptophan to indole-3-lactate and then to indole and skatole [48].

A bidirectional association between the metabolome and the microbiome in the rumen exists and is fundamental for goat production and health. Many significant correlations were found between the altered rumen metabolites and microbiota, such as *Enterobacteriaceae*, *Lactobacillaceae*, and *Prevotellaceae* in goats supplemented with HT and *Halomonadaceae*, *Lactobacillaceae*, and *Prevotellaceae* in those supplemented with CT. *Enterobacteriaceae* are perhaps the single most well-studied family of bacteria, because they include the gut commensal *Escherichia coli*, which has been used as a model organism for more than 135 years [49]. Gheibipour et al. [50] reported that *Enterobacteriaceae* can be isolated from the rumen of rams grazing on poor forages with high tannin, and these bacteria possessed cellulose and protease activities, in addition to potent tannase, which catalyzes the hydrolysis of ester bonds present in gallotannin, complex tannins, and gallate esters to release gallic acid [51]. *Lactobacillaceae* are an essential part of the microbiota in the gut and are mainly composed of the genus *Lactobacillus*, which has the ability to ferment carbohydrates anaerobically into lactate and is commonly used as a probiotic to protect the host against pathogens and stimulate the immune system [52]. Prevotellaceae are prevalent within the rumen and gastrointestinal tract of herbivores and omnivores. They are capable of utilizing starches, other non-cellulosic polysaccharides, and simple sugars as energy sources to produce succinate, which is the major fermentation end product [53]. In addition, *Lactobacillaceae* and *Prevotellaceae* could catabolize tryptophan and tyrosine to produce indole-3-propionic acid, which has a protective factor against metabolic disorders in the host [54]. As ubiquitous, versatile chemoheterotrophs, *Halomonadaceae* can utilize carbohydrates, amino acids, polyols, and hydrocarbons as sole sources of carbon and energy [55], and they were also demonstrated to be associated with glutamate metabolism, which may be involved in excitatory neurotransmission and alterations in serotonin production, thus triggering a cascade of molecular events, including feed intake and the immune regulation [56].

## 5. Conclusions

Based on the findings from this study, it was concluded that dietary supplementation with HT or CT variably modulated the rumen microbiota composition and metabolic profiles to affect growth performance in goats. Among the two types of tannins studied, CT increased feed intake without adverse effects on nutrient digestibility and rumen fermentation when included at 0.5% in the diet. The rumen bacteria Bacteroidetes increased in both HT- and CT-fed goats, while the abundances of Verrucomicrobia and *Methanobrevibacter* decreased after feeding a CT diet. Metabolomics analysis indicated that HT and CT differentially altered the rumen metabolite profiles. Levels of dihydrouracil, xanthosine, alpha-D-mannose, and L-tryptophan decreased with dietary CT supplementation, and these metabolites are related to nucleic acid synthesis, mannose metabolism, and tryptophan metabolism. This study provides insights into the microbial and metabolic mechanisms in goats fed HT and CT diets and may be helpful in facilitating the application of tannins in ruminant production. Nevertheless, the precise mechanism by which HT and CT modulate rumen microbial communities and metabolic profiles, as well as their subsequent correlations with growth performance in goats, remains unclear and warrants further investigation.

## Figures and Tables

**Table 1:** Ingredients and nutritional level of the basal diet (dry matter basis).

Items	Contents	Items	Contents
Ingredients	Level (%)	Nutrients ^2^	Level (%)
Alfalfa	40.00	DM	89.43
Peanut straw	35.00	DE, MJ/kg	9.83
Corn	13.25	CP	14.25
Soybean meal	6.25	NDF	42.72
Rapeseed meal	0.50	ADF	29.24
Corn gluten meal	1.00	Ca	1.00
DDGS	1.75	TP	0.46
Liquid molasses	0.75	Ash	8.52
Limestone	0.50	EE	3.91
Bentonite	0.25		
Premix ^1^	0.30		
Sodium Bicarbonate	0.25		
Sodium chloride	0.20		
Total	100.00		

DM, dry matter; DE, digestible energy; CP, crude protein; NDF, neutral detergent fiber; ADF, acid detergent fiber; Ca, calcium; TP, total phosphorus; EE, ether extract. ^1^ Per kg diet provides: Fe, 21,300 mg; Cu, 18,600 mg; Mn, 16,000 mg; Zn, 58,600 mg; I, 900 mg; Se, 350 mg; Co, 270 mg; VA, 2,000,000 IU; VD_3_, 6,000,000 IU; VE, 40,000 mg; ^2^ DE value was calculated, while other values were measured.

**Table 2:** Effects of dietary supplementation with different types of tannins on the growth performance of Liaoning cashmere goats.

Items	Control	Tannin Groups		SEM	*p*-Value
		HT	CT		
Initial BW, kg	32.36	32.84	31.78	1.24	0.928
Final BW, kg	35.91	36.61	35.87	1.21	0.987
BW gain, kg	3.55 ^b^	3.44 ^b^	4.03 ^a^	0.12	0.008
ADFI, g/d	990 ^b^	908 ^c^	1031 ^a^	17.10	<0.01
F/G, g/g	11.79	11.30	11.29	0.35	0.814

BW, body weight; ADFI, average daily feed intake; F/G, feed intake/body weight gain ratio; SEM, standard error of mean. ^a,b,c^ Means in a row with different superscript letters differ significantly at *p* < 0.05.

**Table 3:** Effects of dietary supplementation with different types of tannins on nutrient digestibility of Liaoning cashmere goats.

Items	Control	Tannin Groups		SEM	*p*-Value
		HT	CT		
DM, g/kg	822.41	815.63	816.18	7.20	0.839
CP, g/kg	718.02	758.54	723.09	10.81	0.960
NDF, g/kg	689.13	644.13	638.37	18.90	0.506
ADF, g/kg	565.14	534.30	515.32	13.09	0.760

DM, dry matter; CP, crude protein; NDF, neutral detergent fiber; ADF, acid detergent fiber; SEM, standard error of mean.

**Table 4:** Effects of dietary supplementation with different types of tannins on rumen fermentation of Liaoning cashmere goats.

Items	Control	Tannin Groups		SEM	*p*-Value
		HT	CT		
pH	6.81 ^a^	6.43 ^b^	6.75 ^a^	0.53	0.003
NH_3_-N, mg/dL	7.10	10.10	9.60	0.05	0.050
Total VFA, mM	74.69	70.78	65.84	3.09	0.521
Molar, %					
Acetic acid	66.46 ^a^	63.53 ^b^	65.92 ^a^	0.61	0.046
Propionic acid	17.27	16.36	15.87	0.44	0.164
Butyric acid	12.09	12.59	12.70	0.28	0.597
Isobutyric acid	2.22	1.87	2.44	0.13	0.218
Valeric acid	0.74 ^b^	1.02 ^b^	1.63 ^a^	0.66	0.042
Isovaleric acid	0.79	1.06	1.93	0.27	0.226
Acetic/Propionic	3.91	4.27	4.57	0.16	0.265

SEM, standard error of mean. ^a,b^ Means in a row with different superscript letters differ significantly at *p* < 0.05.

**Table 5:** Effects of dietary supplementation with different types of tannins on alpha-diversity indices of ruminal microbiota in Liaoning cashmere goats.

Items	Control	Tannin Groups		SEM	*p*-Value
		HT	CT		
Chao1	5897	8866	7713	395	0.059
Shannon	10.00	10.54	10.39	0.18	0.460
Simpson	0.99	0.90	0.99	0.01	0.345

SEM, standard error of mean.

**Table 6:** Effects of dietary supplementation with different types of tannins on the relative abundance of ruminal microbiota at the phylum level in Liaoning cashmere goats (%).

Items	Control	Tannin Groups		SEM	*p*-Value
		HT	CT		
Bacteroidetes	75.18 ^b^	81.39 ^a^	82.54 ^a^	1.76	0.017
Firmicutes	14.78	12.94	11.32	1.31	0.192
Verrucomicrobia	6.35 ^a^	6.84 ^a^	3.67 ^b^	0.87	0.032
Others	3.44	2.82	2.47	0.27	0.174

SEM, standard error of mean. ^a,b^ Means in a row with different superscript letters differ significantly at *p* < 0.05.

**Table 7:** Effects of dietary supplementation with different types of tannins on the relative abundance of ruminal microbiota at the genus level in Liaoning cashmere goats (%).

Items	Control	Tannin Groups		SEM	*p*-Value
		HT	CT		
*Prevotella*	30.98	43.97	38.76	2.13	0.065
*Rikenellaceae* RC9	21.87	18.07	22.29	1.33	0.053
*Bacteroidales F082*	20.37	15.88	17.23	2.52	0.064
*Ruminococcaceae* UCG	11.57	7.30	8.89	0.85	0.115
*Kiritimatiellae WCHB1-41*	7.18	7.49	4.04	0.96	0.096
*Christensenellaceae* R7	1.54	0.38	1.42	0.07	0.403
*Lachnospiraceae* UCG	0.72	0.76	0.69	0.14	0.212
*Candidatus Saccharimonas*	0.69	0.74	0.82	0.60	0.582
*Bacteroidales* RF16	0.22 ^b^	1.25 ^a^	0.75 ^b^	0.34	0.041
*Bacteroidales* BS11	0.38 ^b^	0.15 ^b^	1.54 ^a^	0.06	0.027
*Halomonas*	0.32	0.18	0.22	0.04	0.064
*Succiniclasticum*	0.21	0.17	0.09	0.04	0.276
*Erysipelotrichaceae* UCG	0.18	0.23	0.12	0.19	0.064
*Streptococcus*	0.15	0.07	0.04	0.29	0.198
*Mollicutes* RF39	0.04	0.16	0.14	0.03	0.160

SEM, standard error of mean. ^a,b^ Means in a row with different superscript letters differ significantly at *p* < 0.05.

**Table 8:** Effects of dietary supplementation with different types of tannins on the relative abundance of ruminal methanogen in Liaoning cashmere goats (%).

Items	Control	Tannin Groups		SEM	*p*-Value
		HT	CT		
*Methanobrevibacter*	90.75 ^a^	93.60 ^a^	76.28 ^b^	6.70	0.030
*Candidatus Methanoplasma*	5.92 ^b^	3.68 ^b^	15.06 ^a^	4.67	0.030
*Methanosphaera*	0.33	0.50	0.14	0.24	0.460
*Methanomicrobium*	0.47	0.13	0.11	0.21	0.480
*Candidatus Methanomethylophilus*	0.96	0.81	1.80	0.73	0.370
Unclassified	1.23	0.96	1.71	1.30	0.640

SEM, standard error of mean. ^a,b^ Means in a row with different superscript letters differ significantly at *p* < 0.05.

## Data Availability

The original contributions presented in this study are included in the article/Supplementary Materials. Further inquiries can be directed to the corresponding authors.

## Supplementary Materials

The following supporting information can be downloaded at: https://www.mdpi.com/article/10.3390/microorganisms13040933/s1, Figure S1: Principal component analysis (PCoA) of rumen microbiota from the control and HT groups in Liaoning cashmere goats; Figure S2: Principal component analysis (PCoA) of rumen microbiota from the control and CT groups in Liaoning cashmere goats; Figure S3: Genera responsible for the main differences in rumen microbiota composition duo to supplementation with different types of tannins in Liaoning cashmere goats, detected by linear discriminant analysis (LDA) effect size (LEfSe); Table S1: Effects of dietary supplementation with different types of tannins on ruminal metabolite profiles in Liaoning cashmere goats.
