# Supplementary material for: Microbiomic and Metabolomic Insights into the Roles of Hydrolysable Versus Condensed Tannins on the Growth Performance, Nutrient Digestion, and Rumen Fermentation in Liaoning Cashmere Goats
Source: Microorganisms. 2025 Apr 17;13(4):933. doi: 10.3390/microorganisms13040933 (PMC12029424; doi:10.3390/microorganisms13040933)
Supplement: Supplementary file 1 [file microorganisms-13-00933-s001.zip › microorganisms-3539806-supplementary.pdf]

Table S1. Effects of dietary supplementation with different types of tannins on ruminal metabolite profiles in Liaoning cashmere goats

Compounds	VIP	*p*-value	Log2FC	Regulation
HT vs. Control				
Isovaleric acid	2.43	0.041	0.28	up
L-Valine	1.39	0.017	-0.13	up
Citrulline	2.62	0.030	1.46	up
Theophylline	1.89	0.043	0.22	up
Mannitol	1.45	0.023	0.52	up
all-trans-Retinoic acid	2.02	0.015	-1.63	up
Glycocholic acid	2.07	0.041	0.11	up
Xanthosine	1.53	0.038	0.06	up
Dihydrouracil	1.23	0.015	-0.51	up
L-Tryptophan	3.56	0.025	-0.90	up
alpha-D-Mannose	1.70	0.037	0.20	up
Acetylcholine chloride	1.72	0.015	0.16	down
Kynurenic acid	1.17	0.031	0.51	down
10-Deoxymethynolide	2.17	0.013	0.06	down
L-3-Phenyllactic acid	1.54	0.030	-0.26	down
9,10-Dihydroxystearate	1.06	0.048	-0.24	down
Fomepizole	1.13	0.029	-0.70	down
4-Aminocatechol	1.11	0.015	-0.23	down
Pimelic acid	2.11	0.002	1.23	down
N-Acetylhexosamine	1.47	0.049	-0.49	down
Luteolin	1.02	0.023	0.08	down
(+)-7-Isojasmonic acid	2.05	0.008	-1.26	down
11-Dehydrocorticosterone	1.83	0.025	-1.29	down
CT vs. Control				
L-Valine	1.01	0.019	-0.19	up
2-Hydroxybenzaldehyde	1.15	0.048	-0.06	up
Creatine	2.46	0.026	-0.44	up
2,6-Dihydroxyphenylacetate	1.99	0.018	0.27	up
Pyridoxine	1.26	0.030	0.17	up
D-Lyxose	2.59	0.023	0.13	up
Theophylline	1.07	0.039	-0.63	up
Pelargonic acid	1.19	0.006	0.37	up
D-Alanyl-D-alanine	1.45	0.023	-0.15	up
Citric acid	1.05	0.015	-0.24	up
Mannitol	1.77	0.004	-1.13	up
N1-Acetylspermidine	1.12	0.041	-0.56	up
Isoelemicin	1.50	0.025	-0.69	up
3-Methyl-L-tyrosine	1.33	0.041	-0.53	up
Vanillylmandelic acid	1.73	0.025	-1.31	up

N-Acetyl-D-glucosamine	1.65	0.025	-0.95	up
N-Acetylhexosamine	1.89	0.004	-3.26	up
Pantothenol	1.31	0.041	-0.98	up
beta-Alanyl-L-lysine	1.84	0.004	-1.40	up
N5-Phenyl-L-glutamine	2.08	0.002	-1.64	up
1-Hexadecanol	1.77	0.015	-2.26	up
9-OxoODE	1.49	0.025	-0.72	up
10-Deoxymethynolide	1.192	0.008	-0.95	up
Enterolactone	1.61	0.025	0.66	up
Sphingosine	2.03	0.004	-1.61	up
8,11,14-Eicosatrienoic acid	1.40	0.025	-1.52	up
9,10-Dihydroxystearate	1.78	0.015	-0.67	up
S-Adenosylmethionine	1.49	0.025	-1.83	up
Sodium deoxycholate	1.77	0.004	-1.72	up
Fomepizole	2.06	0.002	-1.91	up
Dihydrouracil	1.69	0.025	-1.33	down
3-Hydroxybenzoic acid	1.57	0.025	-1.30	down
D-(+)-Glucose	1.90	0.015	-1.28	down
Alpha-D-Glucose	1.56	0.041	-1.41	down
alpha-D-Mannose	1.78	0.008	-1.03	down
L-Gulose	1.58	0.041	-0.71	down
Acetylcholine chloride	1.83	0.015	-2.06	down
Sorbitol	1.70	0.041	-0.75	down
L-Tryptophan	1.69	0.015	-0.71	down
12-Hydroxydodecanoic acid	1.62	0.025	-0.53	down
(6Z)-Octadecenoic acid	1.26	0.041	-1.24	down
Xanthosine	1.94	0.002	-2.98	down
Luteolin	1.54	0.041	-0.75	down
11-Dehydrocorticosterone	2.04	0.002	-1.94	down
Nicotinamide ribotide	1.79	0.025	-0.92	down
D-Maltose	2.39	0.002	-4.20	down
D-Lyxose	1.89	0.039	-0.37	down
p-Octopamine	1.06	0.028	0.01	down
2-Keto-glutaramic acid	3.28	0.039	-0.11	down
L-3-Cyanoalanine	2.07	0.030	-1.11	down
2,3-Butanediol	1.34	0.026	-0.30	down
2,3-Butanediol	2.60	0.029	0.28	down
Normetanephrine	1.03	0.041	-0.16	down
3-Dehydroshikimate	2.11	0.030	-0.06	down

VIP, variable importance in projection.

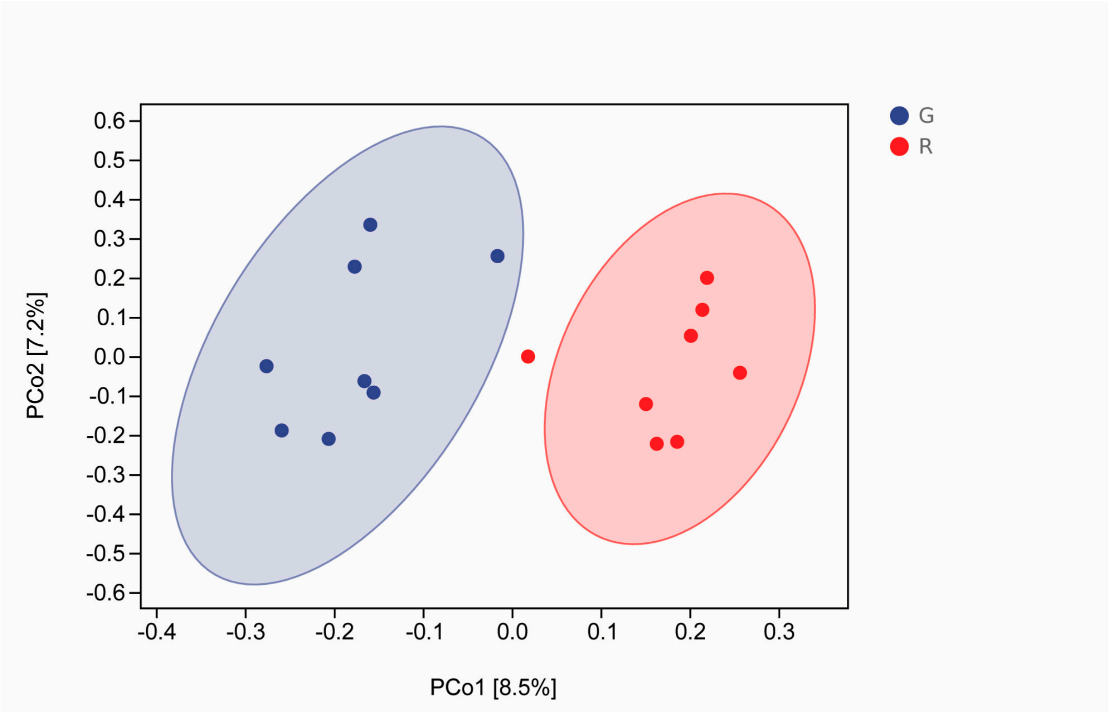

Figure S1. Principal component analysis (PCoA) of rumen microbiota from the control and HT groups in Liaoning cashmere goats. G, control group; R, HT group.

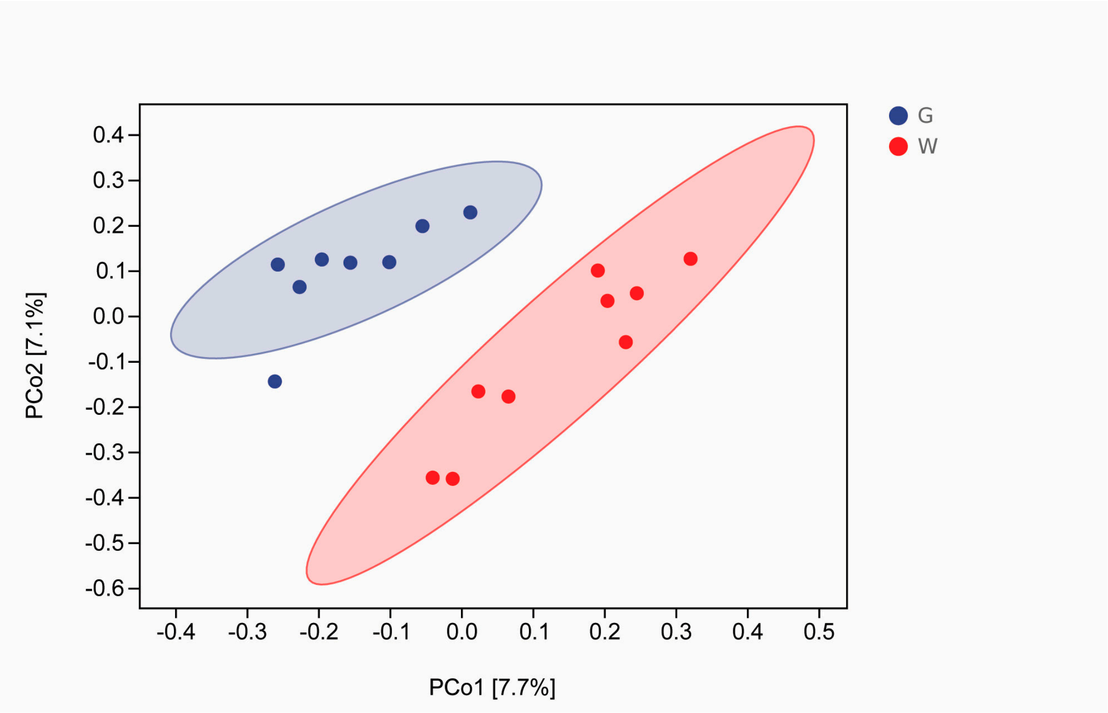

Figure S2. Principal component analysis (PCoA) of rumen microbiota from the control and CT groups in Liaoning cashmere goats. G, control group; W, CT group.

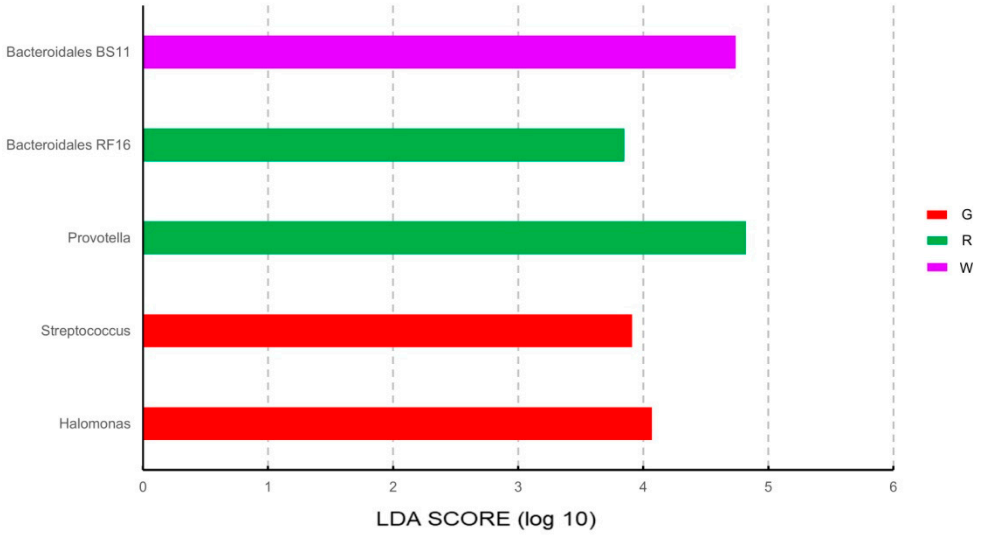

Figure S3. Genera responsible for the main differences in rumen microbiota composition duo to supplementation with different types of tannins in Liaoning cashmere goats, detected by linear discriminant analysis (LDA) effect size (LEfSe). G, control group; R, HT group; W, CT group.
